# Fisheries Bycatch as an Inadvertent Human-Induced Evolutionary Mechanism

**DOI:** 10.1371/journal.pone.0060353

**Published:** 2013-04-10

**Authors:** Christophe Barbraud, Geoffrey N. Tuck, Robin Thomson, Karine Delord, Henri Weimerskirch

**Affiliations:** 1 CEBC-CNRS, UPR 1934, 79360 Villiers en Bois, France; 2 CSIRO Wealth from Oceans Flagship, Division of Marine and Atmospheric Research, Hobart, Tasmania, Australia; Norwegian Polar Institute, Norway

## Abstract

Selective harvesting of animals by humans can affect the sustainability and genetics of their wild populations. Bycatch - the accidental catch of non-target species - spans the spectrum of marine fauna and constitutes a harvesting pressure. Individual differences in attraction to fishing vessels and consequent susceptibility to bycatch exist, but few studies integrate this individual heterogeneity with demography. Here, we tested for the evidence and consequences of individual heterogeneity on the demography of the wandering albatross, a seabird heavily affected by fisheries bycatch. We found strong evidence for heterogeneity in survival with one group of individuals having a 5.2% lower annual survival probability than another group, and a decrease in the proportion of those individuals with the lowest survival in the population coinciding with a 7.5 fold increase in fishing effort in the foraging areas. Potential causes for the heterogeneity in survival are discussed and we suggest that bycatch removed a large proportion of individuals attracted by fishing vessels and had significant phenotypic and population consequences.

## Introduction

Harvesting can change the demographics of wild populations of animals [1], [2], and the evolution of heritable traits [3]–[5]. There is increasing evidence that changes to key demographic parameters and physical attributes (e.g. reduced body size, earlier sexual maturity, reduced antler size) that have been observed over time in exploited populations are due to human-induced evolution caused by selection against desirable phenotypes through harvest [6].

It has increasingly been recognized that individuals of a certain size, morphology or behavior are more likely than others to be removed from a population by harvesting [7], [8]. Among the consistent individual differences in behavioral traits (defined as personality), are exploratory behavior, aggression, and risk taking [9]. Since the landmark study by Wilson *et al.* [10], there is increasing evidence that an animal’s susceptibility to capture is related to personality (e.g. fish: [11], [12]; mammals: [13]; birds: [14]). In harvested populations, heterogeneity in individual personalities may lead to the extinction of vulnerable traits: individuals more susceptible to capture will have increased mortality rates and will progressively disappear from the population [15], [16]. Because of this heterogeneity and because personality traits appear to be heritable [17], selection will occur and the surviving population will differ from the original unharvested population.

However, a major difficulty in examining the evolutionary and ecological consequences of consistent individual personality differences in harvested populations is hidden heterogeneity. Heterogeneity can be modeled with known individual covariates (sex, age, etc.), yet the precise cause of heterogeneity is often not identified, or not measured. To date, studies that related animal personality to life history traits have relied on assessing individual personality through experimental assays where animal scores are coded in standardized tests, and subjective ranking of personality traits are made by human observers [18]. However, unobserved factors at the individual level may generate hidden variability in susceptibility to capture or harvest and can lead to population-level patterns that are not always representative of the actual relationship at the individual level [15]. For example, consider a population consisting of two types of members in equal numbers which differ in values of both survival from harvesting and survival from natural mortality (i.e. heterogeneity in survival), and that there is no compensatory mortality in each subpopulation. Then, Johnson *et al.* [16] demonstrated that if both subpopulations are not distinguished one might infer that the population partly compensates for harvesting mortality by reduced natural mortality. Thus, heterogeneity in survival rates among members of two subpopulations of a harvested population could give the appearance of compensation despite an absence of compensation within each subpopulation.

When the individuals of a species or population are not the primary target of harvesting but constitute incidental catch, they are termed ‘bycatch’. Bycatch spans the spectrum of marine fauna, including fish, seabirds, marine mammals, turtles and benthic invertebrates [19] and constitutes a harvesting pressure. Although some progress has been made in estimating the impact of harvesting non-target species, the demographic, evolutionary and population-level consequences of bycatch remain relatively understudied [20]. Given the heterogeneity of the behavior of individuals in wild populations, some individuals may be more susceptible to bycatch and more likely to be removed from the populations.

The heterogeneity in susceptibility to human-induced mortality, either through harvest or bycatch, is a form of selection. In this study, we test this hypothesis by investigating the evidence for heterogeneity in survival at the individual level in a population of a seabird species, the wandering albatross *Diomedea exulans*, which is heavily affected by bycatch in fisheries [21]. Although we use this species as a case study, the approach is quite general and can be applied to other study situations and taxa. We also model the dynamics of a population of wandering albatrosses, taking into account this hidden heterogeneity, and compare it with conventional models based on estimates of breeding population size from observed count data.

### Background and Predictions

Wandering albatrosses from Possession Island, in the Crozet Islands within the southern Indian Ocean, forage from sub-Antarctic to sub-tropical waters while breeding [22], and extend their range from South Africa to New Zealand while not breeding [23]. The foraging areas of this population overlap with the fishing areas of demersal longliners targeting Patagonian toothfish *Dissostichus eleginoides* in sub-Antarctic waters, and major pelagic longline fisheries targeting southern bluefin tuna *Thunnus maccoyii* and albacore *Thunnus alalunga* in temperate and sub-tropical waters [24]. Individuals foraging at sea may be caught in longlines [25], and bycatch from these fisheries is known to affect adult survival [26] and population dynamics of albatrosses [21], [27].

Although there is evidence that the increase in longline fishing effort (and associated bycatch) from the 1960s to the mid-1980s in the southern Indian Ocean probably increased adult mortality and decreased breeding population size, wandering albatrosses from Possession Island have increased in numbers since the mid-1980s, despite very high levels of longline fishing effort in their foraging areas. In addition, age- and stage-structured population models explicitly modeling bycatch poorly fitted the observed data from the Crozet Islands [27]. This conundrum cannot be explained by the implementation of mitigating measures to reduce albatross bycatch, since (i) these measures were not adopted until the late 1990s and compliance was low in the first years of implementation [28], (ii) mitigation would have affected only the part of the fisheries that overlapped with the foraging areas of wandering albatrosses [28], (iii) albatross bycatch mortality remained high in tuna longline fleets in the southern Indian Ocean where similar, although less numerous, mitigation measures were implemented [29], and (iv) large Illegal Unreported and Unregulated (IUU) longline fishing fleets were operating from the mid-1990s until the mid-2000s [24], [28]. Therefore the increase in the population of wandering albatrosses at Possession Island, and at other breeding sites in the southern Indian Ocean, remains paradoxical [30], [31]. Our aim was to test the hypothesis that hidden heterogeneity in susceptibility to accidental capture (and mortality) by longlines may partly explain this paradox.

Based on the observation that within a population of a given seabird species some individuals appear to be more attracted to fishing vessels than others [32], including albatrosses [33], [34], we hypothesize that this held for our study population of albatrosses, and can account for the paradoxical population trend. The population is assumed to be heterogeneous, with two types of individuals that reflect behavioral syndromes (animal personalities): those strongly attracted by fishing vessels and therefore susceptible to capture and mortality by longlines; and those less attracted by fishing vessels and therefore less susceptible to capture. However, neither the risk-taking or risk-avoiding behaviors can be measured because risk-taking individuals are likely to have been removed and no longer available in the population to measure these traits. From this hypothesis we make the following predictions.

### Prediction 1

If heterogeneity to attraction and susceptibility to capture and accidental mortality by longlines is present in the study population, models explicitly accounting for heterogeneity in survival with two categories of individuals should better predict the survival data than models with only one category of individuals. We thus predict selection of models including two categories of individuals, with one category characterized by a lower survival than the other.

### Prediction 2

If prediction 1 is verified, and given the assumed higher susceptibility of attracted individuals to mortality in longline fisheries and the observed increase in fishing effort through time, we expect the proportion of the category of individuals with the lowest survival to decline and the proportion of individuals of the other category to increase through time. Eventually, once all the individuals of the category with the lowest survival are removed from the population, the proportion of individuals of the other category would remain relatively stable, and if all individuals from the category with the lowest survival are removed then those left would only be individuals from the other category. In addition, the decrease in the proportion of individuals from the category with the lowest survival should coincide with the increase in fishing effort in the foraging area.

## Materials and Methods

### Ethics Statement

Research conducted was approved by the ethic committee of Institut Paul Emile Victor (IPEV) and by the Comité de l’Environnement Polaire.

### Study Site and Population

Wandering albatrosses are large (≈10 kg), long-lived seabirds that breed on sub-Antarctic Islands. We chose to study the wandering albatrosses from Possession Island (46°S, 52°E), Crozet, south-western Indian Ocean, for this particular study because of the extensive and high quality dataset from a long-term monitoring program. The number of breeding pairs was relatively stable during the 1960s, but there was a marked decline between the early 1970s and 1986, followed by an increase until 2003 [35]. From 2003 to 2010, the breeding population has declined slightly.

### Data

From 1960, adults and chicks were ringed with stainless steel rings and since 1966 a capture-mark-recapture program has been undertaken annually between December and April. Most rings of breeding birds were checked in January and February, just after laying, and all chicks were ringed in September and October just before fledging. At fledging, breeding performance was determined for most individuals. Breeding individuals (i.e. those attending a nest) were classified as successful breeders (SB) when they fledged a chick, or failed breeders (FB) otherwise. However, during the early years of the study, the breeding performance of some individuals was not ascertained; they were classified as breeders (B). Each year, new individuals found in the colony were ringed. We used data on breeding adults identified from the 1960 through 2010 breeding seasons, which were either ringed as adults or as chicks. The data were coded with one digit per year: 0 = not observed, 1 = seen as a FB, 2 = seen as a SB, 3 = seen as a B (which were used to build capture histories). This yielded a total of 4339 individual capture histories for a period of 50 years. A restricted data set only including individuals of known sex was also used to test our predictions. Individuals were sexed from plumage characteristics and size. This yielded a total of 1876 female and 1998 male life histories.

Foraging areas were identified from information on the at-sea distribution of breeding and non-breeding adults obtained by telemetry from 1989 to 2010 (satellite tracking using Argos PTT satellite transmitters, global positioning system, and geolocator). Numerous longline fleets operate in the Southern Ocean and are known, or suspected, to interact with Crozet wandering albatrosses and other seabirds. Focusing on the Indian Ocean fleets, because the Crozet wandering albatross population breeds in the southern Indian Ocean and breeding birds forage in the Indian Ocean [22], the fleets with the greatest overlap with the foraging distribution and therefore the greatest potential interactions are the high-seas pelagic longline fleets of Japan and Taiwan [26], [27]. Annual fishing effort was calculated from monthly reported fishing effort data (numbers of hooks deployed) in 5 by 5 degree spatial blocks obtained from the Indian Ocean Tuna Commission (IOTC).

### Model Description and Goodness-of-fit

Our approach was based upon multi-event capture-mark-recapture models [36]. The observer records events [(i) not seen, (ii) seen as a FB, (iii) seen as a SB, (iv) seen as a B] that carry uncertain information on the state of the individual at the current sampling occasion. The relationship between states and events is probabilistic; hence these models belong to the family of hidden Markov models [36].

To take into account the quasi-biennial breeding behavior of wandering albatrosses, and breeding state uncertainty when estimating demographic parameters, we used the approach developed by [37]. In brief, models are described by considering the vector of probabilities of initial presence in the various states, then linking states at successive sampling occasions by a survival-transition probability matrix, and linking events to states by an event probability matrix. Transition probabilities between states were modelled with a three-step procedure where survival, breeding and success were considered as three successive steps in the transition matrices. This baseline multi-event model developed by [37] considers four events (0 = not observed, 1 = seen as a FB, 2 = seen as a SB, 3 = seen as a B), and five states (FB = failed breeder, SB = successful breeder, PFB = post-failed breeder, PSB = post-successful breeder, and dead). Post-failed and post-successful breeder states account for those individuals that skip breeding and remain unobservable at sea in the year following a breeding attempt. Only birds in the FB or SB states are observable, whereas birds in the PFB and PSB states are unobservable.

To accommodate heterogeneity, two categories of individuals were built, each category being associated with a distinct value of the parameter(s) [38], [39]. Because our main predictions concern the effect of heterogeneity on the initial proportions and survival of individuals, the two categories of individuals were built only for these parameters. Thus, in models with heterogeneity on these parameters there will be a state with low survival for one category of individuals and a state with high survival for the other category of individuals and different proportions for both categories. We thus considered an initial model with four events as defined above, and nine states (FB1 = failed breeder category 1, FB2 = failed breeder category 2, SB1 = successful breeder category 1, SB2 = successful breeder category 2, PFB1 = post-failed breeder category 1, PFB2 = post-failed breeder category 2, PSB1 = post-successful breeder category 1, PSB2 = post-successful breeder category 2, and dead). The most general model we considered included heterogeneity in proportions and survival, but not on other parameters.

Several constraints were made to ensure that the model reflected the life-cycle of wandering albatross and did not contain redundant parameters [37]. Encounter and state determination probabilities were time-dependent, and survival probability, breeding probability and success probability were time-independent. Thus our initial model was denoted as (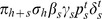) where the subscript *h* refers to heterogeneity in the proportion of newly encountered individuals (denoted *π* and thereafter called initial proportion) and survival (denoted *σ*) probabilities, subscript *s* refers to state dependency in proportion, breeding (denoted as *β*), success (denoted as *γ*), encounter (denoted as *p*) and state determination (denoted as *δ*) probabilities, and superscript *t* refers to time.

To test for linear and quadratic temporal trends over time in initial proportions of individuals in observable states we fitted models where the initial proportion varied according to a quadratic (or linear) trend on a logit scale as:
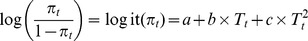
where *a* is the intercept, *b* and *c* respectively the slopes of the linear and quadratic trend terms on the logit scale. This formula was applied to all states simultaneously to ensure that the initial proportion estimates summed to 1.

Models were selected with AIC [40]; the lowest AIC-model was preferred. We used program E-SURGE 1.8.5 [41] to obtain maximum likelihood estimates of the parameters and to select the model. For the restricted data set including sexed individuals, model selection and goodness-of-fit was performed on each sex separately.

The assessment of goodness-of-fit (GOF) is an open question with multi-event models and with models with unobservable states [36]. We therefore made approximate GOF tests on observation histories where the event B (breeding performance not ascertained) was assigned to the SB state (i.e. we assumed that all B events were SB events; [37]). We followed [42] by discounting the change in deviance (Δ*dev*) between models that did not account for unobservable states and models that accounted for unobservable states, state uncertainty and heterogeneity. In this case, the GOF tests were approximated as:
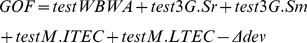
with



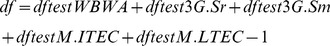



GOF was tested with the program U-CARE 2.5 [43].

### Model Performance

To assess model performance we compared the observed counts of breeding pairs with model-based estimates of the number of breeding pairs obtained from a matrix population model [44]. The deterministic matrix population model was formulated as for the closely related Amsterdam albatross *Diomedea amsterdamensis* [45]. Briefly, this pre-breeding census model consists of five juvenile age-classes, one pre-breeding stage-class and four stage-classes according to breeding status (corresponding to the FB, SB, PFB and PSB states). The model parameters were the recruitment probability, adult survival probabilities, transition probabilities between states, breeding success for first-time breeders, and juvenile survival. Recruitment probability and juvenile survival were estimated from multistate capture-recapture data following [46]. Briefly, we used a multistate model with two states: one immature state and one adult state starting at first reproduction for all those individual ringed as chicks. Our starting model was denoted as (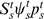) where *S*, *ψ* and *p* are respectively the probabilities of survival, transition between states, and detection, subscript *s* refers to state dependency, and superscript *t* refers to time. The probability of recruitment was described by the transition from immature to adult state. Several constraints were made to ensure that this model reflected the life-cycle of wandering albatross and did not contain redundant parameters. The immature state is assumed to be unobservable since birds ringed as fledglings are never seen again as immature. Thus detection probability for the immature state was fixed at zero. In addition, recruitment never occurred before 5 years of age so that between age two and age four the detection probability of the adult state and the transition probability from the immature to the adult breeding state were fixed at zero. By definition, the local apparent immature survival was estimated over the 5 years of the immature period, and the transition from adult to immature was fixed at zero. We used the same model selection procedure and program as those used for the multi-event model to obtain maximum likelihood estimates of the parameters.

Adult survival and transition probabilities were directly estimated from our multi-event capture-recapture model. Fecundity was modeled as the product of breeding success for successful breeders (by definition equal to 1) and the probability of juvenile survival. We first ran a matrix population model with year-specific adult survival probabilities without heterogeneity obtained from the corresponding multi-event model without heterogeneity. We then ran a matrix model with year-specific adult survival probabilities estimated in presence of heterogeneity and taking into account the initial proportions of individuals in the four observable states as:
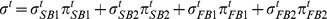
where *t* indicates year, 1 and 2 indicates the two categories of individuals. All other parameters were constant, except for juvenile survival, which was year-specific. Matrix population models were run with the package *popbio* [47] implemented in program R [48]. Initial stage abundances were set equal to the stable age distribution based on the total number of breeding females of 1968.

## Results

The approximate GOF tests indicated that our general multi-event model with unobservable states, state uncertainty and heterogeneity fitted the data (total *χ* ^2^ = 182.9, total *df* = 1014, *P* = 1.00). This was also verified for the restricted data set (males: *χ* ^2^ = 173.3, *df* = 815, *P* = 1.00; females: *χ* ^2^ = 373.0, *df* = 745, *P* = 1.00).

**Figure 1:**
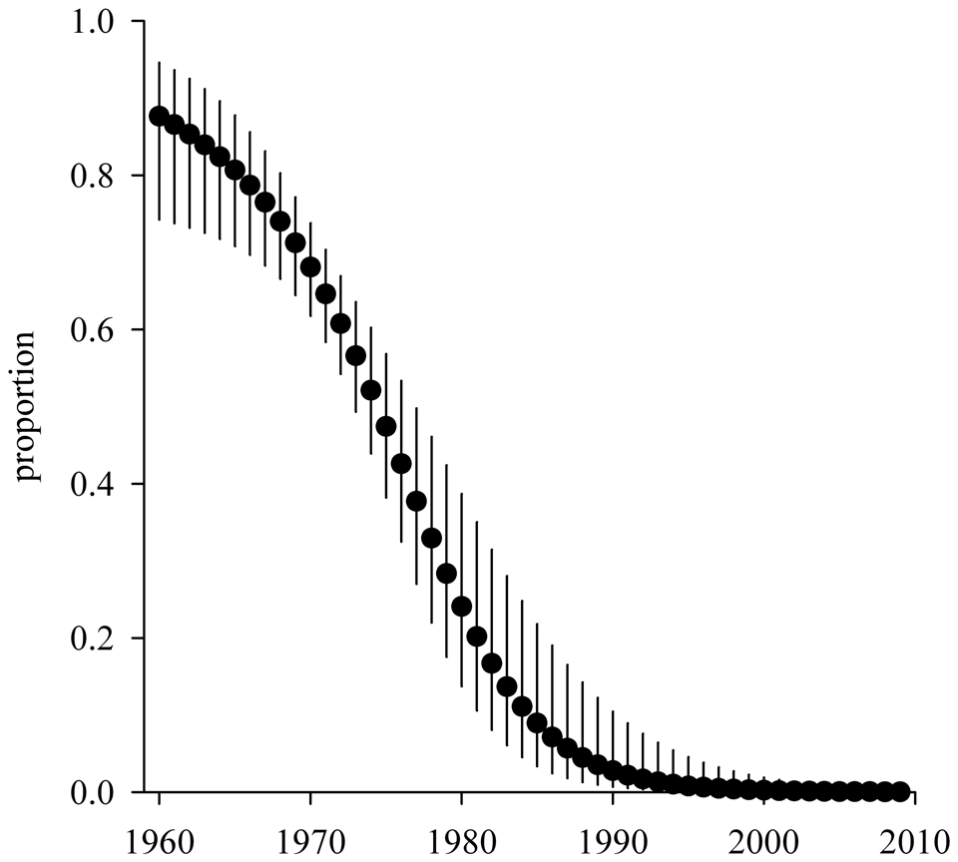
Changes in the proportion of newly encountered individuals (successful breeders) from category 1 in the population of wandering albatrosses from Possession Island between 1960 and 2010. Parameter estimates are from Model 2. Errors bars are 95% confidence intervals.

There was strong support for a model with a linear temporal trend in the proportion of both categories of newly encountered individuals in the population (Table 1). This model (Model 2) was 243 AIC-points lower than Model 1 (constant proportions) and eight AIC-points lower than Model 3 (quadratic trend). Model 2 clearly suggested a decrease in the initial proportion of one category of individuals (category 1) through time and an increase in the initial proportion of the other category of individuals (category 2). This pattern was particularly marked for successful breeders, which constitute the majority of the breeding population (Fig. 1).

**Table 1:** Modeling the effect of heterogeneity and time on survival and initial proportions of two categories newly encountered individuals wandering albatross at Possession Island.

Model	Hypothesis on σ	Hypothesis on π	dev	rank	AIC	def
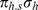 (1)	heterogeneity	heterogeneity	73218	190	73602	0
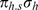 (2)	heterogeneity	heterogeneity and linear trend	72967	194	73359	0
(3)	heterogeneity	heterogeneity and quadratic trend	72967	198	73367	0
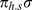 (4)	no heterogeneity	heterogeneity and linear trend	73214	193	73600	0

The candidate models vary in the presence/absence of heterogeneity on survival (σ) and of temporal trends on survival and proportions (π). For all models breeding and success probabilities were state dependent and constant, and encounter and state assignment probabilities were state and time-dependent. For each model the deviance (dev), rank, AIC and ΔAIC are given. Subscripts *h* and *s* refer to heterogeneity and state, respectively, *T* to a linear temporal trend and *T+T^2^* to a quadratic temporal trend. def indicates rank deficiency.

**Figure 2:**
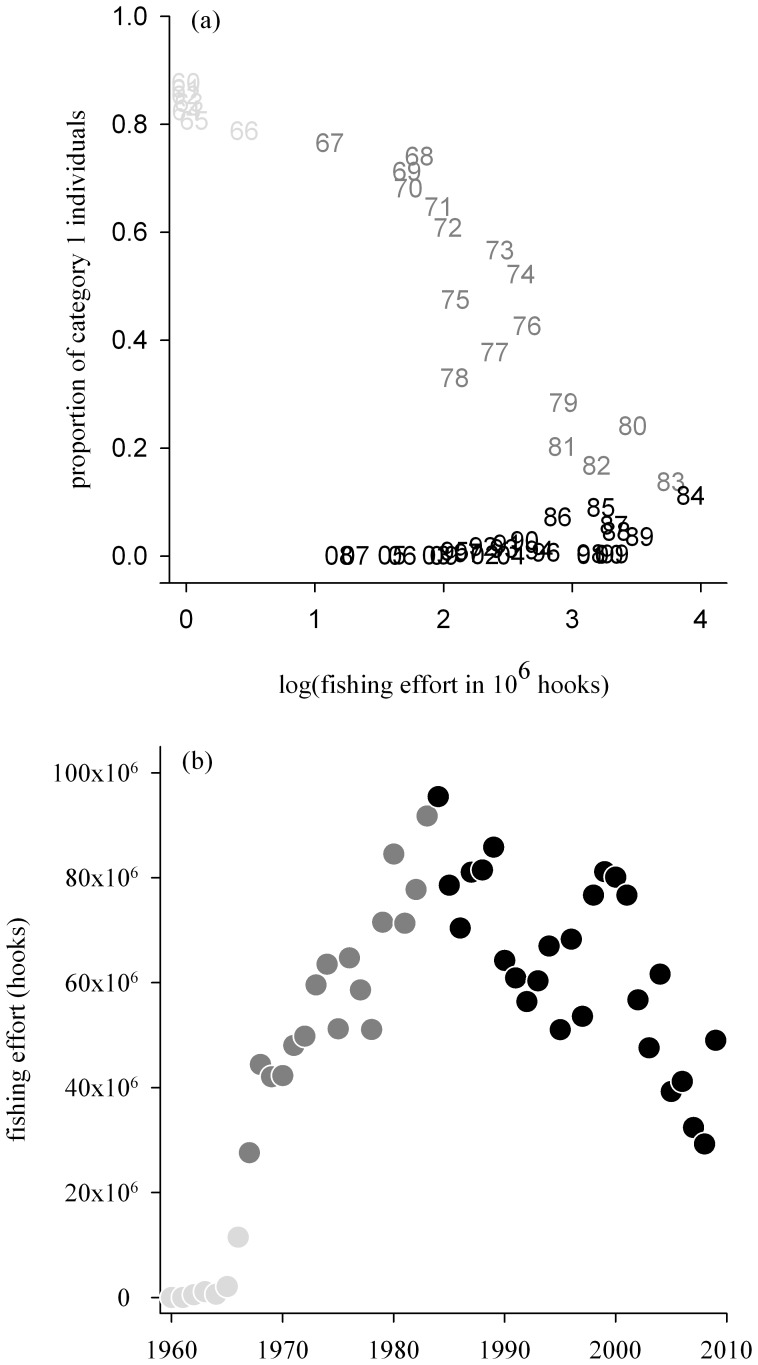
Proportion of newly encountered individuals (successful breeders) from category 1 and longline fishing effort. Changes in (a) the proportions of category 1 individuals in the population of wandering albatrosses from Possession Island between 1960 and 2010, as a function of longline fishing effort south of 30°S in the Indian Ocean; and (b) the annual estimated longline fishing effort south of 30°S in the Indian Ocean for the Japanese and Taiwanese fisheries combined. Parameter estimates in (a) are from Model 2. Light grey, dark grey and black coding correspond to time periods of low, increasing and decreasing fishing effort.

Interestingly, the decrease in the initial proportion of category 1 individuals coincided with the increase in fishing effort in the foraging areas between ≈1966 and ≈1990 when effort increased by a factor of 7.5 (Fig. 2). The rate of decrease in the initial proportion of category 1 individuals was particularly high from 1970. From 1990 to 2010 the initial proportion of category 1 individuals has remained low and nearly all newly encountered individuals in the population are classified in category 2.

For annual survival there was strong support for a model with heterogeneity. A model with no heterogeneity in survival (Model 4) was 241 AIC-points lower than Model 2. Estimates from Model 2 indicated that survival of category 1 individuals was 5.2% lower (mean ± SE = 0.900±0.004) than survival of category 2 individuals (0.949±0.002).

Over the dataset there was strong evidence for linear trends over time in the initial proportions of both categories of newly encountered individuals and for heterogeneity in adult survival. The same model structure (Model 2) was retained for both sexes as for the entire dataset (Table 2), suggesting that the above processes were also operating in males and females. The amount of individual heterogeneity in survival seemed more reduced in females than in males (category 1 males: 0.936±0.003; category 2 males: 0.962±0.002; category 1 females: 0.938±0.004; category 2 females: 0.943±0.003), but overall male and female average survival did not differ (males: 0.947±0.003; females: 0.938±0.004).

**Table 2:** Modeling the effect of heterogeneity and time on survival and initial proportions of two categories newly encountered individuals for males and females wandering albatross at Possession Island.

Model	Hypothesis on σ	Hypothesis on π	dev	rank	AIC	def
Males						
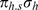 (1)	heterogeneity	heterogeneity	38880	190	39268	0
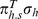 (2)	heterogeneity	heterogeneity and linear trend	38819	194	39214	0
(3)	heterogeneity	heterogeneity and quadratic trend	38810	198	39214	0
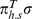 (4)	no heterogeneity	heterogeneity and linear trend	38878	193	39271	0
Females						
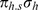 (1)	heterogeneity	heterogeneity	35301	190	35688	0
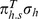 (2)	heterogeneity	heterogeneity and linear trend	35282	194	35677	0
(3)	heterogeneity	heterogeneity and quadratic trend	35288	198	35693	0
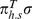 (4)	no heterogeneity	heterogeneity and linear trend	35302	193	35696	0

The candidate models vary in the presence/absence of heterogeneity on survival (σ) and of temporal trends on survival and proportions (π). For all models breeding and success probabilities were state dependent and constant, and encounter and state assignment probabilities were state and time-dependent. For each model the deviance (dev), rank, AIC and ΔAIC are given. Subscripts *h* and *s* refer to heterogeneity and state, respectively, *T* to a linear temporal trend and *T+T^2^* to a quadratic temporal trend. def indicates rank deficiency.

Using the entire dataset, we built an *a posteriori* model with heterogeneity on breeding and success probabilities. This model was 273 AIC-points lower than Model 2, strongly suggesting the presence of heterogeneity in breeding parameters. *Post hoc* comparisons between traits indicated significant heterogeneity in breeding probability for successful breeders in the previous year and in success probability in failed breeders in the previous year (Table 3).

**Table 3:** Mean (SE) survival, breeding and success probabilities of both categories of newly encountered individuals.

Parameter	Category 1	Category 2	*P*
Σ	0.9085 (0.0034)	0.9463 (0.0025)	<0.001
β_FB_	0.8935 (0.0116)	0.9221 (0.0131)	0.102
β_SB_	0.0196 (0.0072)	0.0684 (0.0045)	<0.001
β_PFB_	0.0803 (0.0158)	0.1717 (0.0818)	0.273
β_PSB_	0.9999 (0.0001)	0.9934 (0.0168)	0.699
γ_FB_	0.3236 (0.0307)	0.6548 (0.0157)	<0.001
γ_SB_	0.7349 (0.0164)	0.7250 (0.2536)	1.000
γ_PFB_	0.1734 (0.3853)	0.3721 (0.0187)	0.606
γ_PSB_	0.5029 (0.0197)	0.5362 (0.1398)	0.813

Parameter estimates from a model with the same structure as Model 2 (Table 1), but with heterogeneity in breeding and success probabilities. Tests to compare parameters between both categories of individuals were performed with program Contrast [58].

**Figure 3:**
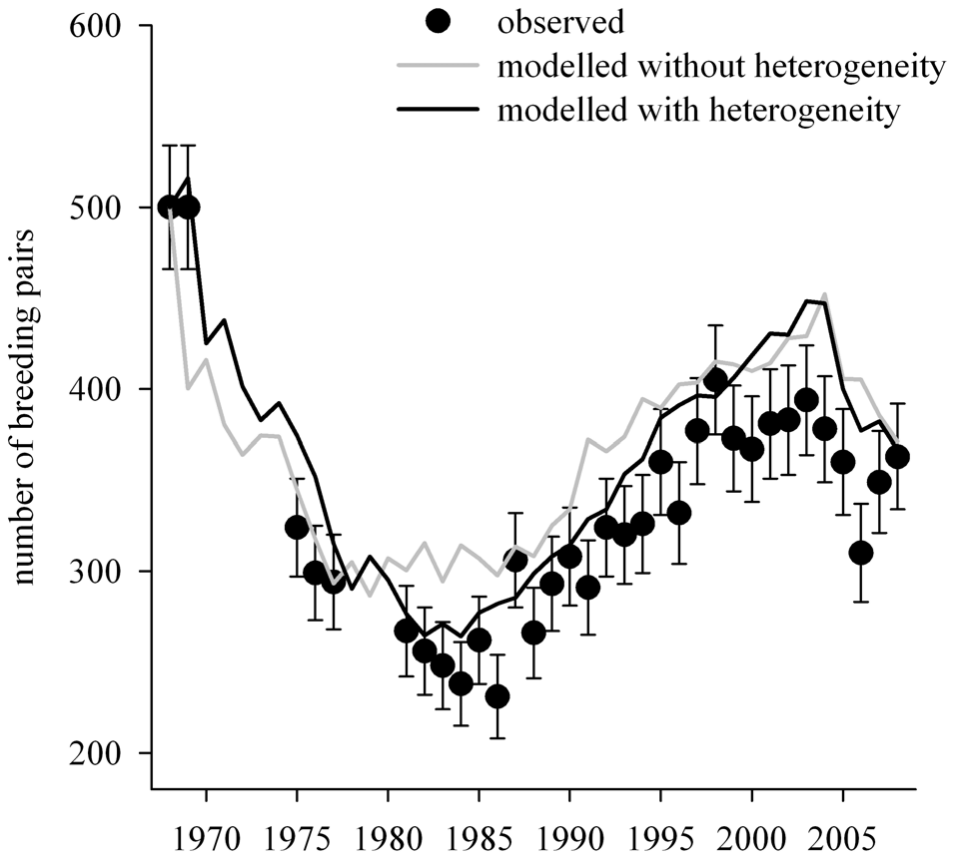
Numbers of breeding pairs of wandering albatrosses at Possession Island, from 1968 to 2008. Black dots indicate observed counts (error bars are ± SE), grey line indicates numbers predicted by a matrix population model without heterogeneity on adult survival, and black line indicates numbers predicted by a matrix population model with heterogeneity on adult survival.

The deterministic matrix population model taking into account heterogeneity in survival better predicted the observed counts of breeding pairs (linear regression: *r* ^2^ = 0.89, *P*<0.001) than the matrix population model that ignored this heterogeneity (*r* ^2^ = 0.72, *P*<0.001, Fig. 3). Population growth rates were 0.968 for the category 1 and 1.007 and for the category 2 subpopulations, indicating respectively a 3.2% annual decrease and a 0.7% annual increase. The generation time for the category 1 subpopulation was 19 years, whereas for the category 2 subpopulation it was 25.4 years.

## Discussion

We found strong evidence for heterogeneity in survival in a wandering albatross population heavily affected by bycatch in longline fisheries. As predicted under the hypothesis of differential vulnerability to bycatch, models taking into account heterogeneity fitted the data better (both capture-recapture and population data) than models ignoring heterogeneity. One category of individuals had a 5.2% lower adult annual survival rate than the other category of individuals, which is considerable for a species with such a long generation time (≈21 years, estimated from [44] p.129).

Consistent with our second prediction, the estimated initial proportion of category 1 individuals decreased through time from an initial value of ≈0.87 in the early 1960s (whereas the initial proportion of category 2 individuals in the population increased through time). These trends were consistent with population growth rates that can be estimated from the specific survival probabilities of the population subsets of both categories of individuals using matrix models (Fig. 3). Remarkably, the decrease of category 1 individuals coincided with the increase in fishing effort in the foraging area of this population, although the models used for estimating the initial proportions of both categories of individuals were not constrained by fishing effort. The decrease mainly occurred between ≈1966 and ≈1990, corresponding well with the ≈7.5 fold increase in fishing effort during this period. Thereafter, the initial proportion of category 1 individuals remained low.

These results are congruent with the hypothesis of some individuals in this population of wandering albatrosses (those belonging to category 1) being more likely than others to be killed by longlines, and that when longline fishing effort greatly increased they disappeared from the population. The observed heterogeneity in survival may correspond to two types of individuals that reflect behavioral syndromes, such as those strongly attracted by fishing vessels and therefore susceptible to capture and mortality by longlines, and those less attracted by fishing vessels and less susceptible to capture. Indeed, recent studies showed that some individuals appear to be more attracted to fishing vessels than others on a handful of seabird species [32], including albatrosses [33], [34]. Harvesting, fishing or trapping can produce within-species differential vulnerability in target species [6], [12], [14]. Our results suggest that the proportion of low-surviving individuals among all new breeders has declined dramatically over time. Newly-encountered individuals in our dataset are mainly those individuals born in the study population and returning to breed for the first time (i.e. new recruits). Indeed, immigration (and emigration) is very low in this population [49] and most adults were ringed at the beginning of the study. Consequently, assuming that the observed heterogeneity in survival corresponds to two types of individuals, we speculate that fisheries bycatch selectively removed some individuals from this wild population according to their susceptibility to being caught incidentally, and mainly during the immature period between fledging and first reproduction. This is coherent with the observation that most wandering albatrosses caught in longlines in the Australian Fishing Zone were immature [50], and with the mean age of individuals from our study population caught in longline fisheries targeting tuna (4.7+2.6 years, *n* = 9).

However, we are aware that there are other possible explanations for heterogeneous survival. Sex can be excluded as a potential explanation, since we found evidence for heterogeneity in survival and linear trends in initial proportion of newly encountered individuals in both sexes, and sex differences in average survival are negligible during the time period considered here ([37] and above results). Nevertheless heterogeneous survival linked to morphological differences independent of gender (which could not be tested here due to insufficient data) could potentially influence at-sea distribution and therefore the likelihood of interacting with different fisheries, with implications for mortality. Additionally heterogeneous survival can also originate from genetic differences in personalities [51] which may or may not be correlated to the behavior of individuals relative to the fishing vessels, or from heterogeneity in individual quality and/or the conditions experienced during early life or previous reproductive attempts [52].

Recent studies suggest that some personality traits are implicated in demographic and evolutionary changes in harvested populations [12], [53]. Our results suggest that the differential vulnerability of individuals to incidental capture can also have consequences for the evolutionary dynamics of populations. First, it helps to explain why this and other populations of wandering albatrosses, which decreased in the early 1960s, have been increasing since the mid-1980s, despite longline fishing effort remaining high [21], [30]. Second, it also possibly explains the increase of the closely related Amsterdam albatross over the past decades [45], despite an extensive overlap between its foraging areas and longline fishing activities in the southern Indian Ocean [54]. We suspect that Amsterdam albatrosses vulnerable to fisheries bycatch may have been removed from the population when fishing effort increased in the late 1960s, and that only individuals less attracted by fishing vessels and therefore less susceptible to capture remained in the population, enabling the population to increase. Overall, this suggests that within-species variation in vulnerability to being caught by longliners might affect the population’s response to incidental mortality in fisheries. Third, the selective removal of individuals as bycatch may have induced changes in life history traits. For example, when heterogeneity in breeding success probability was introduced in the multi-event models, the breeding success probability of category 2 individuals that failed in the previous year was significantly higher than those of category 1 individuals that failed in the previous year (0.655±0.016 vs. 0.324±0.031, respectively). Therefore one may hypothesize that the increasing initial proportion of category 2 individuals in the population may have contributed to the temporal increase in breeding success observed at the population level [15], [55]. Finally, some studies have shown that vulnerability of fish to angling is a heritable trait and is related to parental behaviors (e.g. [11], [56]). Although estimating heritability to the vulnerability of being caught as bycatch is challenging in wild populations, recent developments in telemetry combined with long-term studies of known individuals may shed some light on heritability of behavioral interactions between fishing vessels and species affected by bycatch.

We suspect that our results could apply to a large number of species affected by bycatch, since bycatch acts as a harvesting pressure whereby individuals of a certain size, morphology or behavior are more likely than others to be removed from a population by harvesting [12], [14]. Few studies have explored the differential susceptibility to bycatch of individuals in natural populations of animals. The importance of personality to harvest was shown by, for example, Biro & Post [12] in a whole-lake experiment, where the greater harvest of fast-growing individual rainbow trout *Oncorhynchus mykiss* was attributed to their greater behavioral vulnerability: they are more active and bold, i.e. risk taking. In another study, Wilson *et al.* [52] highlighted relationships between individual differences in behavior and capture technique in bluegill sunfish *Lepomis macrochirus*: fish caught by angling were more timid than fish caught with a seine net.

Our study gives circumstantial evidence that some individuals may be more vulnerable to longlines than others. However, we recognize that one limitation of our study is that we could not identify behavioral and/or phenotypic characteristics associated with the degree of vulnerability to fisheries bycatch of individual wandering albatrosses. At present we can only formulate hypotheses, for example, that since some individuals are consistently more attracted to fishing boats, they therefore are more likely to attempt to catch baits on hooks attached to longlines and to risk being killed [32]–[34]. Other hypotheses could be that all individuals are attracted to fishing vessels, but some individuals are less skilled in removing baits without being caught, or individuals that are more aggressive when attempting to remove baits are more likely to be killed. These hypotheses will be tested through telemetry, although our results suggest that one category of individuals currently constitute most of the study population, which may impair our ability to detect a contrast in the behavior of individuals. Given the potential ecological and evolutionary importance of selective mortality due to bycatch, further work to identify individual predictors of susceptibility to bycatch is critical to deepen our understanding of this fascinating and important phenomenon. Finally, from a conservation point of view, one can envisage that in species affected by bycatch, differences in the vulnerability of individuals could improve a population’s response to this human-induced mortality [57], and our results support this hypothesis. However, our results also suggest that exposure to bycatch can substantially reduce within-population heterogeneity associated with vulnerability to being caught as bycatch, and affects generation time with potential consequences for population recovery. That could, in turn, reduce the ability of populations to respond to other or new threats.
